# Where to Send Our Students

**Published:** 1877-07-20

**Authors:** 


### WHERE TO SEND OUR STUDENTS.

A number of medical gentlemen have, within the last few weeks, asked our views touching the advantages and facilities of the various medical colleges. In reply, we will say, in our advertising department may be noted the advertisements of a number of medical institutions, with the terms and peculiar facilities of each set forth. We wish to draw no distinction between them, being satisfied that they are all first-class schools. By addressing a note to the dean, or secretary, of any one of them, no doubt full and satisfactory information can be obtained.

We freely accord with the party writing us, from Tennessee, that preceptors having pupils should make timely inquest as to the morals of the schools, and direct their students to colleges having the best facilities and the highest standard of medical education.

